# Density Functional Study of Electrocatalytic Carbon Dioxide Reduction in Fourth-Period Transition Metal–Tetrahydroxyquinone Organic Framework

**DOI:** 10.3390/molecules29102320

**Published:** 2024-05-15

**Authors:** Yufeng Wen, Xianshi Zeng, Yanan Xiao, Wen Ruan, Kai Xiong, Zhangli Lai

**Affiliations:** 1 School of Mathematical Sciences and Physics, Jinggangshan University, Ji’an 343009, China; wenyufeng@jgsu.edu.cn (Y.W.); zengxueliang@163.com (X.Z.); yananxiao06@163.com (Y.X.); ruanwen@jgsu.edu.cn (W.R.); 2 Materials Genome Institute, National Center for International Research on Photoelectric and Energy Materials, School of Materials and Energy, Yunnan University, Kunming 650091, China; xiongkai@ynu.edu.cn; 3 Advanced Computing Center, Information Technology Center, Yunnan University, Kunming 650091, China

**Keywords:** fourth-period transition metals, tetrahydroxyquinone, organic frameworks, CO_2_ electrocatalytic reduction, density functional theory (DFT) calculations

## Abstract

This study investigates the utilisation of organometallic network frameworks composed of fourth-period transition metals and tetrahydroxyquinone (THQ) in electrocatalytic CO_2_ reduction. Density functional theory (DFT) calculations were employed in analysing binding energies, as well as the stabilities of metal atoms within the THQ frameworks, for transition metal TM-THQs ranging from Y to Cd. The findings demonstrate how metal atoms could be effectively dispersed and held within the THQ frameworks due to sufficiently high binding energies. Most TM-THQ frameworks exhibited favourable selectivity towards CO_2_ reduction, except for Tc and Ru, which experienced competition from hydrogen evolution reaction (HER) and required solution environments with pH values greater than 5.716 and 8.819, respectively, to exhibit CO_2_RR selectivity. Notably, the primary product of Y, Ag, and Cd was HCOOH; Mo produced HCHO; Pd yielded CO; and Zr, Nb, Tc, Ru, and Rh predominantly generated CH_4_. Among the studied frameworks, Zr-THQ displayed values of 1.212 V and 1.043 V, corresponding to the highest limiting potential and overpotential, respectively, while other metal–organic frameworks displayed relatively low ranges of overpotentials from 0.179 V to 0.949 V. Consequently, it is predicted that the TM-THQ framework constructed using a fourth-period transition metal and tetrahydroxyquinone exhibits robust electrocatalytic reduction of CO_2_ catalytic activity.

## 1. Introduction

The increasing global demand for energy and the urgent need to address climate challenges have made searching for sustainable energy conversion and storage technologies critical. Traditionally, carbon dioxide (CO_2_) has been considered a valueless waste gas. However, recent studies have demonstrated that CO_2_ can be transformed into valuable chemicals and fuels through CO_2_ reduction, leading to efficient CO_2_ utilisation and reduced greenhouse gas emissions [1,2,3]. The CO_2_ reduction reaction enables the direct production of valuable C_1_–C_3_ chemical products, such as CO, CH_4_, HCOOH, HCOH, CH_3_OH, C_2_H_4_, C_2_H_6_, C_2_H_5_OH, C_3_H_6_, and others, using various pathways, including electrochemical [4,5], chemical reforming [6], photochemical [7], and biochemical [8] processes. Among these pathways, the electrocatalytic reaction stands out due to its favourable reaction conditions and high process cleanliness, making it highly practical. However, the technology of electrocatalytic CO_2_ reduction faces challenges in terms of low efficiency, short catalyst life, and limited yield. Consequently, the design and development of new catalysts with high efficiency and cost-effectiveness is urgently needed to overcome these limitations.

Metal–organic frameworks (MOFs) are crystalline materials that consist of metallic ions bonded to organic molecules by coordination bonding. MOFs possess a tunable and porous structure, offering large specific surfaces and controllable chemical, as well as physical, properties [9,10,11,12,13,14]. These characteristics make MOFs highly suitable for the designing and synthesis of efficient catalytic converters, leading to significant advancements in catalysis research [15,16,17,18,19,20,21,22,23,24]. The tunability of MOFs allows for precise control of catalytic reactions, while their porous structures provide numerous active sites. Additionally, the large specific surface area of MOFs enhances the reaction rate. By carefully selecting and coordinating organic ligands and metal ions, the catalytic activity, stability, and selectivity of MOFs can be fine-tuned. Consequently, MOFs hold significant potential for a wide range of catalysis applications, offering new opportunities for the design of effective catalysis.

In recent years, there has been extensive research into electrocatalysis using metal–organic frameworks (MOFs) in CO_2_ reduction [25,26,27]. Considering the favourable charge transport properties of conductive materials, they exhibit significant advantages in electrocatalysis. Tetrahydroxyquinone (THQ), an organic skeleton material with abundant active sites and a tunable electronic structure, has gained considerable attention as a potential catalyst material. Majidi et al. [28] constructed two-dimensional copper-based conducting MOF nanosheets known as copper–tetrahydroxyquinone (Cu-THQ). These nanosheets demonstrated excellent catalytic activity for CO_2_ reduction using 1 mol/L potassium hydroxide and 1 mol/L choline chloride mixed electrolyte, with a remarkably low overpotential of only 16 mV. At −0.45 V applied voltage (with respect to the reversible hydrogen electrode), electric densities reached approximately 173 mA/cm^2^, and Faraday’s average efficiency for CO production reached around 91%. Notably, at lower overpotentials, the CO generation current densities of this conducting MOF were found to be 35 and more than 25 times higher than the currently reported catalytic current densities of similar MOFs and MOF derivatives, respectively. The significant progress achieved in laboratory experiments has inspired further theoretical exploration of the application of transition metal (TM)-THQ electrocatalysis for CO_2_ reduction. In fact, theoretical research in the field of catalysis has experienced vigorous development [29]. Previous studies have systematically investigated a family of metal–organic TM-THQ materials using density functional theory (DFT) in CO_2_ electrocatalytic reduction reactions [30].

It is notable that in contrast to the third-period transition metals, the fourth-period transition metals exhibit diverse electronic configurations and coordination geometries, thereby offering enhanced versatility in the fabrication of MOFs incorporating THQ. The potential application of these materials as electrocatalysts for CO_2_ reduction remains uncertain, with their effectiveness in this capacity yet to be determined. In light of these considerations, we constructed TM-THQ metal–organic frameworks using fourth-row transitional metal elements and performed a comprehensive investigation into their electrocatalytic performance for CO_2_ reduction. The computational analysis indicates that among 10 monolayers of fourth-period transition metal TM-THQ compounds spanning from Y to Cd, the binding energies between the metal atoms and THQ are substantial enough to enable the stable dispersion of metal atoms within THQ framework. With the exception of Tc and Ru, the remaining TM-THQ compounds demonstrate favourable selectivity for CO_2_ reduction reaction (CO_2_RR), as well as relatively few overpotential sites and high product selectivity. By expanding the application range of TM-THQ metal–organic framework materials in CO_2_ reduction, we aim to provide novel insights and opportunities for a deeper understanding and optimisation of CO_2_ catalytic reduction reactions.

## 2. Calculation Details

All computational work in this study was performed using the Dmol3 software package on the basis of spin-polarised density functional theory [31]. The electronic exchange–correlation interactions were characterised in terms of generalised gradient approximation (GGA) using the Perdew–Burke–Ernzerhof (PBE) functional [32]. We employed the DNP basis set and combined it with the DSPP approximation scheme, replacing core electrons with single effective potentials and introducing corrections for relativistic effects to improve computational efficiency and accuracy [33]. To account for weak long-range interactions between molecules during surface adsorption, we incorporated a van der Waals correction (DFT-D2) [34,35,36,37] for improved description of adduct phenomena. Since the catalytic reactions take place in a watery environment, we adopted a solvation model—the conductor-like screening model (COSMO)—with solute water (relative dielectric constant of ε=78.54) in simulating the effect of the solvent on the system. In view of the eliminating interaction between neighbouring periodic patterns, we included a 25 Å thick vacuum layer. In order to enhance computational accuracy, we set the criterion for energy convergence to 1.0 × 10^−6^ eV and employed 3 × 3 × 1 and 12 × 12 × 1 k-point Monkhorst–Pack grids for structure optimisation and electronic structure calculations, respectively.

To investigate the interaction strength between intermediates and THQ monolayers, we introduced the definition of adsorption energy ($E_{ads}$). Taking the adsorption energy of CH_4_ as an example, it is defined as follows (Equation (1)):

(1) $$E_{\mathrm{ads}}\;=E_{TM-THQ-{\mathrm{CH}}_{4}}-E_{TM-THQ}-E_{{\mathrm{CH}}_{4}}$$

where $E_{TM-THQ-CH_{4}}$ represents the energy of the whole system, where CH_4_ is adsorbed to the surface of TM-THQ; $E_{TM-THQ}$ represents the energy of the TM-THQ monolayer; and $E_{CH_{4}}$ represents the energy of a single molecule of CH_4_. Since CO_2_RR involves multiple reaction pathways, we introduced Gibbs free energy to identify the optimal reaction route. In the case of reactions involving electron transfer, we employed a hydrogen-electrode standard model developed by Nørskov et al. [38,39,40] to calculate reaction energy. The calculation formula for the Gibbs free energy is as follows (Equation (2)):

(2) $$\Delta G=\Delta E+\Delta E_{ZPE}-T\Delta S+\Delta G_{pH}+\Delta G_{U}$$

where ΔE represents the energy of the reaction; $\Delta E_{ZPE}$ and ΔS describe zero-point energy and entropy changes, respectively (experimental values are used for small molecules); *T* represents the reaction thermodynamic temperature (298.15 K); $\Delta G_{pH}$ represents free energy due to differences in acidity/alkalinity (different $H^{+}$ concentrations) and is given by $\Delta G_{pH}=2.303$ $K_{B}T_{pH}$, with pH assumed to be 0 in solutions that are acidic; and $\Delta G_{U}$ is the correction of free energy due to electrode potential differences and is given by Equation (3):

(3) $$\Delta G_{U}=-neU$$

with *n* being the number of electrons transferred and *U* being the potential applied to the electrode. The overpotential (η) and limiting potential ($U_{L}$) are essential parameters in the evaluation of catalyst activity. The limiting potential can be calculated from Equation (4) as follows:

(4) $$U_{L}=-\Delta G_{max}/ne$$

wherein $\Delta G_{max}$ represents free-energy change in the rate-determining step. Equation (5) shows that the difference between the equilibrium potential ($U_{equilibrium}$) and the limiting potential is the overpotential.

(5) $$\eta =U_{equilibrium}-U_{L}$$

Stability is a critical factor in assessing the performance of catalysts. To assess TM-THQ stability, calculations were performed to determine the cohesive energy of bulk metal, the binding energy of TM-THQ, and the formation energy. The cohesive energy ($E_{c}$) is given by Equation (6).

(6) $$E_{c}=\left(E_{M(bulk)}-nE_{M}\right)/n,$$

wherein $E_{M(bulk)}$ and $E_{M}$ represent the bulk and individual metal atom energies, respectively, and *n* represents the number of bulk metal atoms. The energy of binding ($E_{b}$) is then obtained from Equation (7).

(7) $$E_{b}=E_{TM-THQ}-E_{TM}-E_{THQ},$$

in which $E_{TM-THQ}$, $E_{TM}$, and $E_{THQ}$ correspond to energies of the TM-THQ backbone, individual metallic atoms, and ligand (tetrahydroxyquinone), respectively. Additionally, we calculated the formation energies of different MOFs to examine the simplicity with which TM-THQ monolayers could be prepared. The formation energy ($E_{f}$) is calculated as shown in Equation (8).

(8) $$E_{f}=E_{TM-THQ}-n_{TM}\mu_{TM}-n_{C}\mu_{C}-n_{O}\mu_{O},$$

where $E_{TM-THQ}$ represents the energy of TM-THQ, and $n_{TM}$, $n_{C}$, and $n_{O}$ denote the number of metal atoms, carbon atoms, and oxygen atoms, respectively. $\mu_{TM}$, $\mu_{C}$, and $\mu_{O}$ represent transition metal, carbon, and oxygen chemical potentials, respectively, with the chemical potential of metal atoms being an individual metal atom’s energy within the most stable metal crystals, the chemical potential of carbon atoms being the individual energy of carbon in graphite, and the chemical potential of oxygen atoms being half the energy of O_2_.

## 3. Result and Discussion

### 3.1. Analysing the TM-THQ Structure

**Figure 1:**
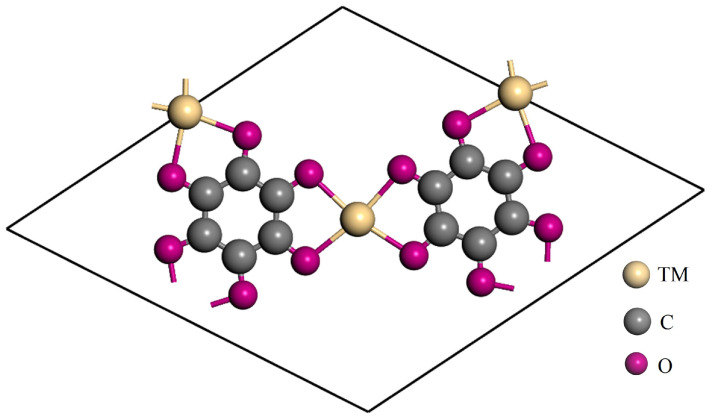
The top view of a single cell of TM-THQ, where metal atoms are marked by yellowish spheres, carbon atoms are indicated by grey spheres, and oxygen atoms are indicated by pink spheres.

Figure 1 illustrates a top-down view of a single cell of a fourth-period transition metal–tetrahydroxybenzoquinone (TM-THQ) metal–organic framework (MOF) material. The image clearly depicts that each single cell consists of 12 carbon atoms (C), 12 oxygen atoms (O), and 3 transition metals. Notably, each metallic atom coordinates with four oxygen bonds within the ligands of tetrahydroxybenzoquinone. Importantly, all ten fourth-period transition metals considered in this study, ranging from yttrium to cadmium, are located within the same plane. Table 1 provides insight on bond lengths from 2.017 Å to 2.345 Å between the metallic and nearest oxygen atoms. Furthermore, to investigate the electronic states of these ten MOF materials and conduct a Hirshfeld charge analysis, Table 1 demonstrates that in the series of the considered fourth-period transition metals, the metal atoms have partial positive charges, while the nearest oxygen atoms exhibit opposite partial negative charges. This suggests a transfer of electrons from metal atoms to the THQ skeleton, leading to both ligand-bonding interactions and ionic-bonding interactions between metal and oxygen atoms. Spin-state analysis of metals reveals that all except molybdenum (Mo) exhibit non-spin states. Molybdenum, on the other hand, displays a spin state with a 2.459 μB magnetic moment.

### 3.2. Stability of TM-THQ

**Figure 2:**
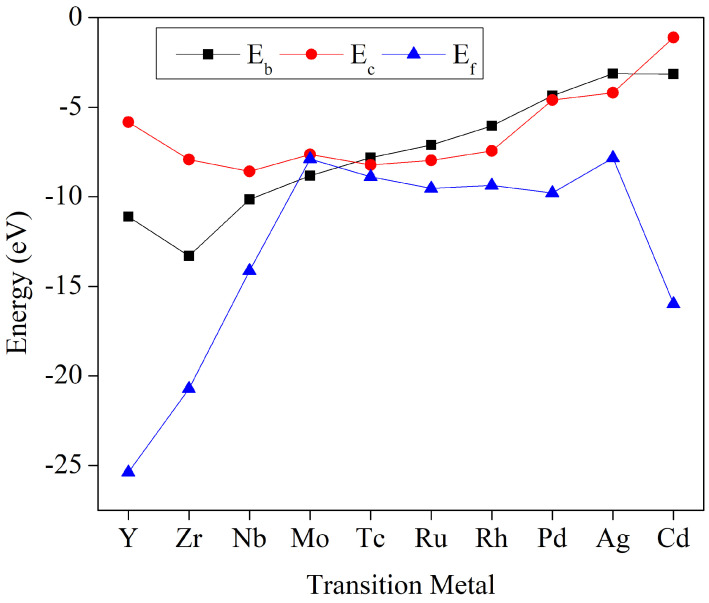
The cohesive energy ($E_{c}$), binding energy ($E_{b}$), and formation energy ($E_{f}$) of TM-THQ catalysts with fourth-period transition metals.

Table S1 provides the binding energetics of the ten TM-THQ catalysts, as well as the corresponding cohesive energies of metals. Figure 2 illustrates the binding energies, formation energies, and cohesive energies of each metal for these ten MOFs. A negative formation energy indicates an exothermic reaction during the preparation process, suggesting easier material synthesis. It is evident that the binding energies of all ten TM-THQ structures are negative, indicating the potential experimental feasibility of synthesising these MOFs. When TM-THQ metal–organic frameworks are synthesised, a stronger bond from metal atoms to the substrate hinders metal atom aggregation, enabling metal atoms to be uniformly and stably embedded in the substrate. The higher cohesion energies of Tc, Ru, Rh, Pd, and Ag compared to their respective TM-THQ structures suggest a propensity for aggregation during the preparation of these five types of TM-THQ skeletons.

### 3.3. Selectivity of TM-THQ Catalytic Converters towards CO_2_ Reduction (CO_2_RR) and Hydrogen Evolution Reaction (HER)

Traditionally, electrocatalytic CO_2_ reduction in solution occurs via multi-electron step processes. Under an external voltage, solution proton–electron pairs ($H^{+}+e^{-}$) gradually participate in reaction. Once the CO_2_ molecules are adsorbed onto the catalyst surface, the subsequent outcome hinges on whether C or O atoms are the ones being adsorbed. In the event of C-atom adsorption, the addition of H to an oxygen atom ($*+CO_{2}+H^{+}+e^{-}\to^{*}COOH$) yields the intermediate *COOH, with * denoting the catalyst surface. Conversely, when O atoms are adsorbed onto the catalyst surface and H is added to the carbon atom ($*+CO_{2}+H^{+}+e^{-}\to^{*}OCHO$), the result is the formation of the intermediate *OCHO. However, it is also possible for hydrogen to be attached to a catalyst metal atom ($*+H^{+}+e^{-}\to^{*}H$), leading to an undesired hydrogen evolution reaction (HER) during CO_2_RR. In fact, CO_2_RR and HER are competing reactions, necessitating consideration of material selectivity towards CO_2_RR and HER when evaluating CO_2_RR catalysts.

**Figure 3:**
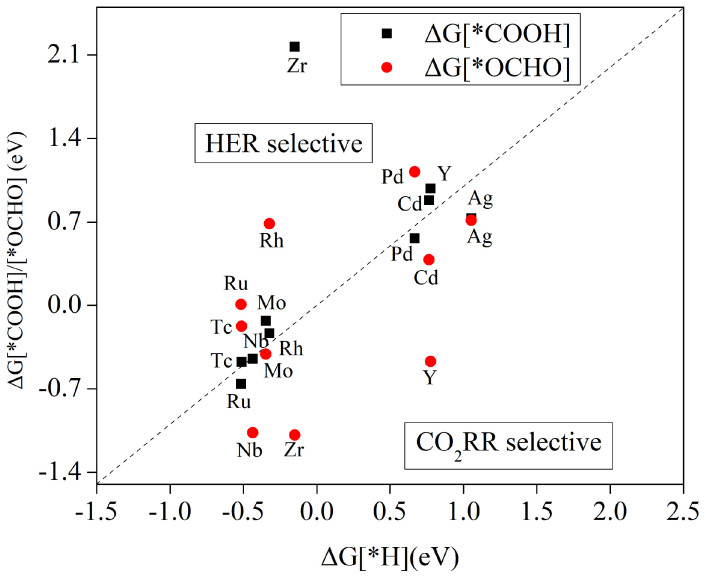
The comparison of CO_2_RR and HER with respect to the Gibbs free energy of protonation during the initial step on the surface of TM-THQ. The position of the catalysts underneath the dotted curve signifies favourable selectivity towards CO_2_RR.

Figure 3 illustrates the variations in Gibbs free energy during initial protonation for the formation of *OCHO, *COOH, and *H species. The values of these parameters can be found in Table S2. It is observed that among the ten investigated transition metals, Tc and Ru catalysts exhibit higher Gibbs free-energy changes compared to the generation towards *H intermediates, irrespective of the formation of *COOH or *OCHO species, indicating a preference for HER reactions. Conversely, the Ag-THQ catalyst demonstrates favourable CO_2_RR activity. For Ru and Pd catalysts, *COOH intermediates dominate, while *OCHO intermediates prevail for Nb, Mo, Zr, Y, and Cd catalysts. Notably, once the active site is occupied by CO_2_ during catalysis, no further *H intermediates can be generated, resulting in CO_2_RR selectivity for these MOF materials.

**Figure 4:**
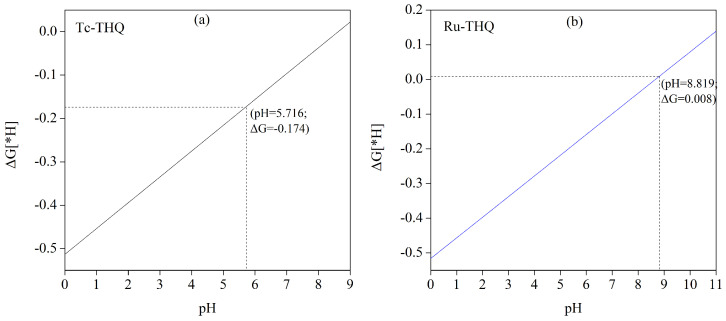
The pH-dependent change in Gibbs free energy of the adsorption process of H to generate *H on Tc-THQ (**a**) and Ru-THQ (**b**) catalysts.

When employing Tc and Ru catalysts for CO_2_ electrocatalytic reduction, it is necessary to adjust the electrolyte solution pH to enhance Gibbs free energy for *H formation. This adjustment inhibits the occurrence of the HER reaction and promotes smooth CO_2_RR. The relationship between the change in Gibbs free energy for *H generation ($\Delta G_{\left[*H\right]}$) and pH can be described by the equation $\Delta G_{pH}=2.303\times K_{B}T\times pH$, wherein Boltzmann’s constant is $k_{B}$, *T* is the reaction temperature (usually 298.15 K), and pH is the pH value of the electrolyte solution. At pH = 0, the $\Delta G_{\left[*H\right]}$ values for Mn are 0.553 eV. These $\Delta G_{\left[*H\right]}$ values exhibit a linear relationship with pH. Figure 4 illustrates the dependence of $\Delta G_{\left[*H\right]}$ on pH for both Tc-THQ and Ru-THQ catalysts in the adsorbed H state. It is observed that the $\Delta G_{\left[*H\right]}$ value increases with increasing pH. Figure 4a shows that when the solution pH is 5.716, Tc-THQ has a $\Delta G_{\left[*H\right]}$ of −0.174 eV. At pH values greater than 5.716, Gibbs free energy for *H formation surpasses the formation energy of *COOH/*OCHO, resulting in CO_2_RR selectivity. It is observed that the $\Delta G_{\left[*H\right]}$ value increases with increasing pH. Similarly, as shown in Figure 4b, Ru-THQ demonstrates CO_2_RR selectivity in solution environments with pH values greater than 8.819.

### 3.4. Potential Pathways and Adsorption Energies

As TM-THQ electrocatalytic carbon dioxide reduction involves a monatomic process, it is recognised that the formation of multicarbon products is challenging due to the inability of monatomic catalysis to facilitate C–C coupling between intermediates. Consequently, from a theoretical perspective, monoatom-catalysed CO_2_ reduction processes are primarily focused on C_1_ products. The most commonly observed C_1_ products in electrocatalytic CO_2_ reduction include CO, CH_4_, HCOOH, CH_3_OH, and HCHO. The intermediate steps and reaction paths corresponding to each electronic step of the catalytic process are shown in Figure S1 [41,42,43,44,45]. The structural models of the intermediates are shown in Table S12.

According to the proposed scheme for electrocatalytic CO_2_ reduction to obtain C_1_ products [41,42,43,44,45], the reduction of CO_2_ to CO and HCOOH involves a 2e process. The paths of reduction can be described as $CO_{2}\to^{*}COOH\to^{*}CO\to CO$ and $CO_{2}\to^{*}OCHO\to^{*}HCOOH\to HCOOH$. In contrast, HCHO production follows a 4e process with reaction pathways $CO_{2}\to^{*}COOH\to^{*}CO\to^{*}CHO\to *OCH_{2}\to HCHO$. Additionally, the production of CH_3_OH requires a 6e process, with pathways described as $CO_{2}\to^{*}COOH\to^{*}CO\to^{*}CHO\to^{*}OCH_{2}\to^{*}OCH_{3}\to^{*}OHCH_{3}\to CH_{3}OH$. The most complex pathway is for the production of CH_4_, which has three possible routes, namely (1) $CO_{2}\to^{*}COOH\to^{*}CO\to^{*}COH\to^{*}C\to^{*}CH\to^{*}CH_{2}\to^{*}CH_{3}\to^{*}+CH_{4}$, (2) $CO_{2}\to^{*}COOH\to^{*}CO\to^{*}CHO\to^{*}OCH_{2}\to^{*}OCH_{3}\to^{*}O+CH_{4}\to^{*}OH\to H_{2}O$, and (3) $CO_{2}\to^{*}COOH\to^{*}CO\to^{*}CHO\to^{*}OCH_{2}\to^{*}OCH_{3}\to^{*}OHCH_{3}\to^{*}OH+CH_{4}\to *+H_{2}O$. Due to the limitations of the electrocatalytic CO_2_ reduction route, to predict the likely product of each catalytic converter, we initially calculated the C_1_ product adsorption energy of the catalyst.

**Figure 5:**
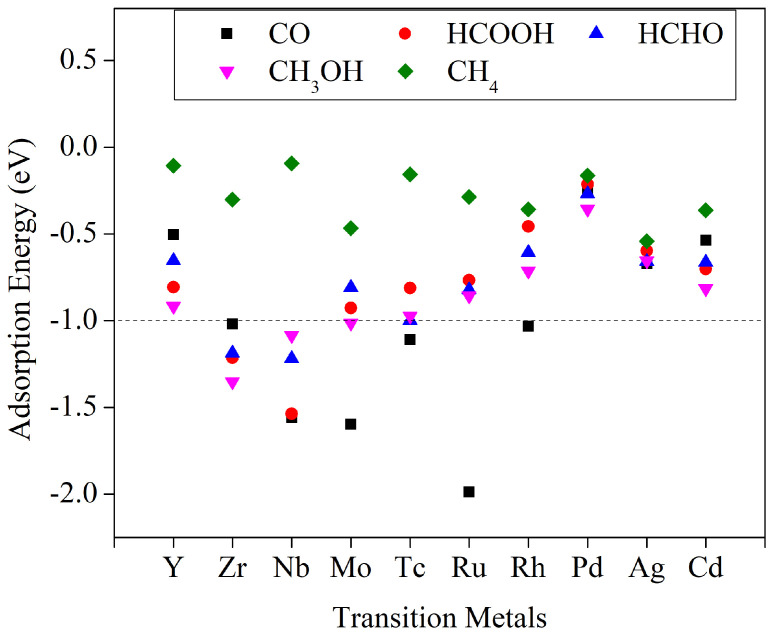
The adsorption energies of various C_1_ products on the surfaces of respective catalysts.

The adsorption energies of the five C_1_ products on the catalyst surfaces are presented in Figure 5, with corresponding values provided in Table S3. Figure 5 reveals that Y, Pd, Ag, and Cd exhibit relatively weak adsorption capacities for the five C_1_ products. Consequently, once these products are generated, they can be easily desorbed from the catalyst surfaces without difficulty. In contrast, Zr and Nb demonstrate relatively strong adsorption energies for CO, HCOOH, HCHO, and CH_3_OH. As a result, these products cannot be generated through desorption from the catalyst surfaces during the catalytic process. Fortunately, their adsorption of CH_4_ is not as strong, allowing the catalytic process to exclusively yield CH_4_ products. Similarly, based on the adsorption energy levels, the catalytic process involving Mo does not require consideration of CO and CH_3_OH as products, Tc does not need to consider CO and HCHO as products, and Rh does not need to consider CO as a product.

### 3.5. Electrochemical CO_2_ Reduction Pathways

#### 3.5.1. Significant Generation of CO Products

**Figure 6:**
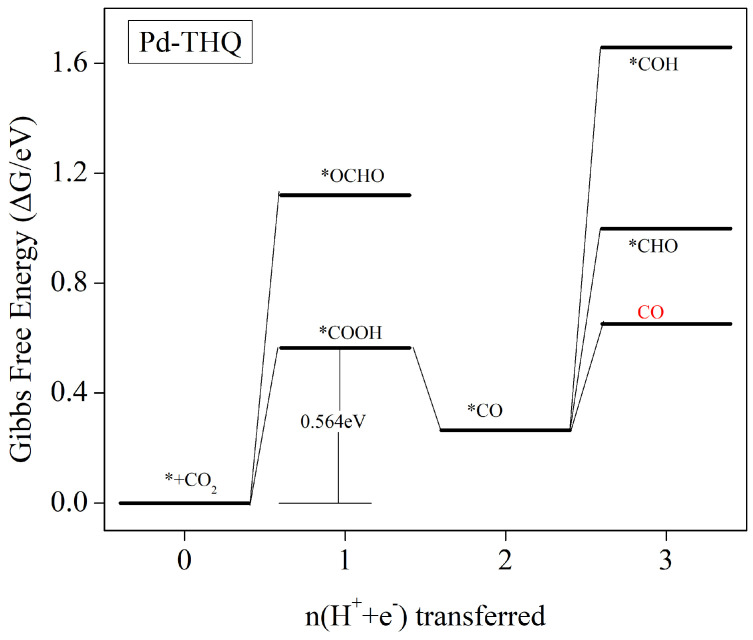
The Gibbs free-energy profile of the CO_2_ reduction reaction (CO_2_RR) at zero potential following the most thermodynamically favourable pathway for Pd-THQ. In this analysis, CO_2_ molecules in the gas phase interacting with a pristine surface are assumed to have a reference free-energy value of zero.

Changes in free energy related to the individual protonation steps in the catalytic process were calculated. It was determined that Pd-THQ’s predominant product in the electrocatalytic reduction of CO_2_ is carbon monoxide (CO).The Gibbs free-energy changes associated with the formation of intermediates during individual protonation steps within the Pd-THQ electrocatalytic CO_2_ reduction process are shown in Figure 6. The corresponding chemical equations with their respective Gibbs free-energy change values can be found in Table S4. In the initial steps in Pd-THQ electrocatalytic CO_2_ reaction, the formation of intermediate *OCHO by protonation exhibits a significantly higher Gibbs free energy than the *COOH intermediate, which represents the initial step in the process. Consequently, the production of *COOH intermediates dominates this step. The subsequent step involves the protonation of *COOH intermediates to form *CO, which is a reduced free-energy exothermic reaction. As a result, *CO intermediates are readily obtained. These *CO intermediates can be further protonated to form *CHO or *COH intermediates, which require overcoming of the 0.734 eV and 1.394 eV energy barriers, respectively, as indicated in Table S4. Moreover, the *CO intermediate can undergo CO desorption, leading to the termination of the catalytic reaction and the generation a final product. This CO desorption step requires overcoming of a 0.387 eV energy barrier, which is lower than that for the generation of *CHO or *COH intermediates. Therefore, the catalytic reaction is primarily terminated through CO desorption in this particular step. Overall, the major product in Pd-THQ electrocatalytic CO_2_ reduction is CO, following the reaction pathway of $*+CO_{2}\to^{*}COOH\to^{*}CO\to CO$. Following the pathway of $*+CO_{2}\to^{*}COOH\to^{*}CO\to CO$, the rate-determining step is $*+CO_{2}\to^{*}COOH$, corresponding to a limiting potential of 0.564 V.

#### 3.5.2. Significant Generation of HCHO Products

**Figure 7:**
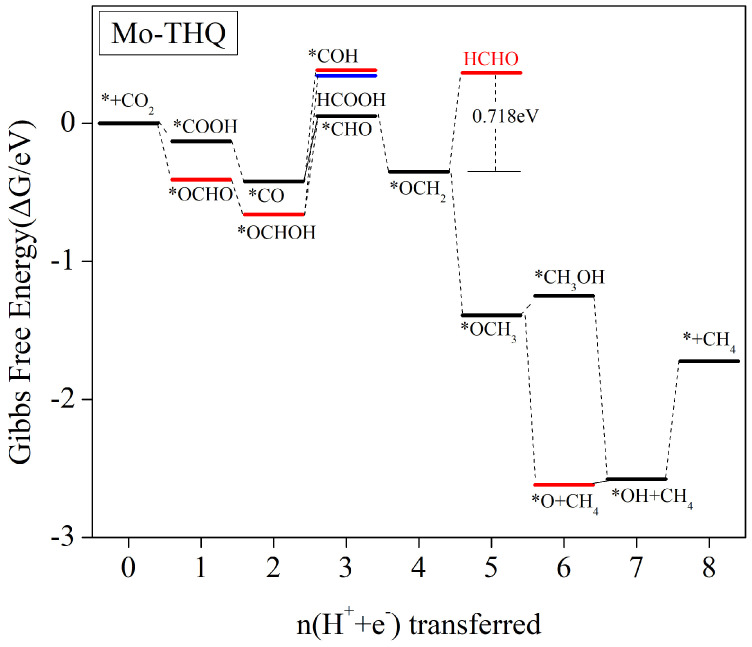
The Gibbs free-energy profile of CO_2_RR along the most favourable pathway for Mo-THQ at zero potential. CO_2_ molecules in the gas phase on a clean surface are assigned zero free energy.

Our investigation revealed that the primary product of the Mo-THQ catalyst in the electrocatalytic CO_2_ reduction process is formaldehyde (HCHO). Figure 7 shows a step-by-step diagram of the Mo-THQ electrocatalytic CO_2_ reduction process. Additionally, comprehensive information regarding changes in Gibbs free energy associated with protonation steps can be found in Table S5. Based on the adsorption energy depicted in Figure 5, the adsorption of carbon monoxide (CO) and methanol (CH_3_OH) by the Mo-THQ catalyst is excessively strong, making it unlikely for these products to be generated through desorption from the catalyst surface. As a result, we did not consider the possibility of producing CO or CH_3_OH as reaction products.

After CO_2_ molecules are adsorbed onto the catalyst surface, all exothermic reactions from $*+CO_{2}\to^{*}COOH{/}^{*}OCHO\to^{*}CO{/}^{*}OCHOH$ proceed with decreasing free energy, facilitating their occurrence. However, in subsequent steps, these reactions may become heat-absorbing with increasing free energy. When considering the generation of HCOOH products, a 1.005 eV energy barrier (Table S5) must be overcome for the ∗OCHOH→∗+HCOOH step, while a 0.475 eV energy barrier (Table S5) must be surpassed for *$CO\to^{*}CHO$, which exhibits the lowest energy among all possible reactions. Consequently, the *$CO\to^{*}CHO$ step primarily leads to the formation of a *CHO intermediate. The subsequent step, *$CHO\to^{*}OCH_{2}$, is an exothermic reaction with reduced free energy. After the formation of the *OCH_2_ intermediate, the *$OCH_{2}\to *+HCHO$ step requires overcoming of an energy barrier of 0.718 eV (Table S5). Simultaneously, further protonation occurs, resulting in the generation of *OCH_3_ as a free energy-reduced exothermic reaction. At first glance, after the formation the *OCH_2_ intermediate, the catalytic reaction is more likely to continue with protonation, where subsequent protonation reactions can either remain exothermic at a lower free energy or require a low energy barrier be overcome. However, the *$OH+CH_{4}\to *+CH_{4}+H_{2}O$ step ultimately requires overcoming of a 0.853 eV energy barrier (Table S5). Hence, the CH_4_ production energy barrier is more challenging to overcome than the HCHO production energy barrier. Throughout the procedure, HCHO is the predominant product obtained. The whole pathway can be summarised as $*+CO_{2}\to^{*}COOH\to^{*}CO\to^{*}CHO\to^{*}OCH_{2}\to HCHO$. The rate-determining step is $*OCH_{2}\to *+HCHO$, corresponding to a limiting potential of 0.718 V.

#### 3.5.3. HCOOH as the Predominant Product of Formation

**Figure 8:**
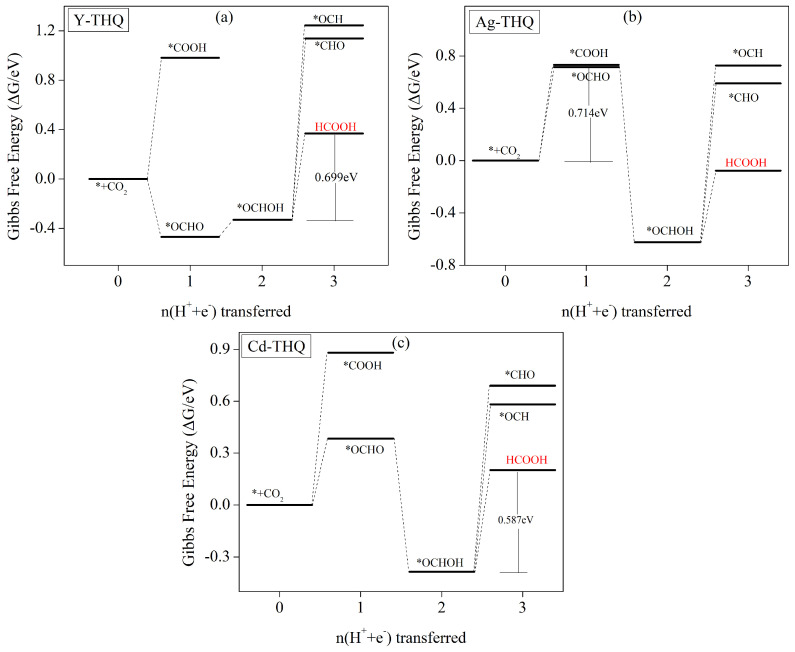
The curves of Gibbs free energy for (**a**) Y-THQ, (**b**) Ag-THQ, and (**c**) Cd-THQ with potential zero, illustrating optimal pathways for the CO_2_ reduction reaction (CO_2_RR). The reference point for free energy is a CO_2_ molecule interacting with a catalytic clean surface in the gas phase.

Computational studies reveal HCOOH to be the primary product in electrocatalytic reduction of CO_2_ using the three investigated catalysts, namely Y-THQ, Ag-THQ, and Cd-THQ. Figure 8 illustrates the energy-step diagrams for the intermediate steps involved in the electrocatalytic processes of these catalysts. Additionally, Table S6 presents the electrochemical equations for the corresponding steps, along with the values of the Gibbs free energies. Figure 8a presents a step diagram of Gibbs free-energy variation for intermediate steps in the electrocatalytic reduction of CO_2_ using the Y-THQ catalyst. Upon CO_2_ molecule adsorption on the catalyst surface, the initial step of protonation to form *COOH exhibits an increase in free energy. However, the subsequent formation of *OCHO is a reaction that is exothermic, with a free energy decrease leading to the formation of *OCHO. The subsequent step of ∗OCHO→∗OCHOH involves crossing a lower energy barrier. Once *CHOH is formed, further protonation occurs to generate *CHO/*COH, both of which require the crossing of a higher energy barrier. On the other hand, HCOOH desorption, which leads to the final product, needs a lower 0.699 eV (Table S6) energy barrier to be crossed. Thus, the reaction concludes with the generation of HCOOH in this step. The overall reaction path can be summarised as $*+CO_{2}\to *OCHO\to *OCHOH\to HCOOH$, with ∗OCHOH→∗+HCOOH as the rate-determining step and 0.699 V as the corresponding limiting potential.

For Ag-THQ and Cd-THQ catalysts, the electrocatalytic CO_2_ reduction process exhibits similarities. Figure 8b,c depict Gibbs free-energy ladders for intermediate processes with these catalysts. Upon CO_2_ adsorption on the surface of the catalyst, the initial step of protonation to form *COOH/*OCHO is an endothermic process with increasing free energies. However, the energy barrier required to generate *OCHO is significantly lower, making it dominant in the competition and eliminating the need to consider the *COOH intermediate-generation step. Subsequently, the *$OCHO\to^{*}OCHOH$ reaction are exothermal, with decreased free energy. Once *OCHOH is produced, the barrier energy needed to stop the reaction by HCOOH desorption is lower. Consequently, both catalysts conclude the reaction by generating HCOOH. The reaction path can be summarised as $*+CO_{2}\to^{*}OCHO\to^{*}OCHOH\to HCOOH$. For Ag-THQ, the rate-determining step is $*+CO_{2}+H^{+}+e^{-}\to *OCHO$, which corresponds to a 0.714 V limiting potential. On other hand, the rate-determining step for Cd-THQ is *OCHOH→∗+HCOOH, corresponding to a 0.587 V limiting potential. Thus, the rate-determining step for both catalysts is *OCHOH→∗+HCOOH, corresponding to a 0.587 V limiting potential.

#### 3.5.4. CH_4_ as Main Catalyst Product

**Figure 9:**
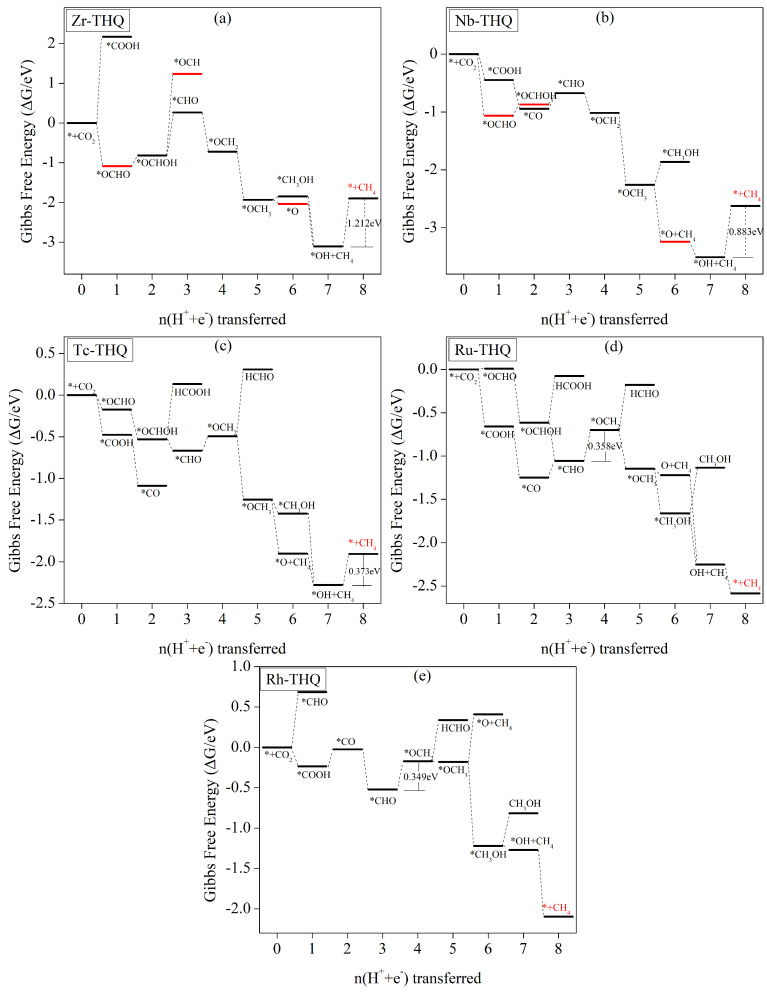
The curves of Gibbs free energy for (**a**) Zr-THQ, (**b**) Nb-THQ, (**c**) Tc-THQ, (**d**) Ru-THQ, and (**e**) Rh-THQ at zero potential along the most favourable path for CO_2_RR. The zero point of free energy is defined as a molecule of CO_2_ in the gas phase with respect to a catalytic clean surface.

Computational studies have revealed that the chemical reduction of CO_2_ by electrocatalysis using Zr, Nb, Tc, Ru, and Rh catalysts produces CH_4_. However, in the case of Zr-THQ catalysts, the surface adsorption of other products, except for CH_4_, is so strong that they cannot be obtained through desorption. Therefore, we only consider the CH_4_ product for Zr-THQ catalysts. Figure 9a illustrates the change in Gibbs free energy of the intermediates in each step of protonation in Zr-THQ electrocatalytic CO_2_ reduction, with corresponding electrochemical equations and values of Gibbs free-energy change presented in Table S7. Upon CO_2_ adsorption on the surface of the catalyst, protonation to form *COOH necessitates the overcoming of a 2.176 eV energy barrier (Table S7). However, generation of *OCHO is a lower-energy exothermic reaction and is therefore the preferred pathway. Subsequently, the further generation of *OCHOH intermediates and their protonation to *OCH/*CHO intermediates involve 2.054 eV and 1.083 eV energy barriers, respectively. Hence, we only consider the route to generate *CHO. The subsequent protonation steps are mostly free energy-reduced exothermic reactions until the 8e process is completed with the production of CH_4_. Thus, CH_4_ is the main product of Zr-THQ electrocatalytic CO_2_ reduction. The reaction pathway can be described as $*+CO_{2}\to^{*}OCHO\to^{*}OCHOH\to^{*}CHO\to^{*}OCH_{2}\to^{*}OCH_{3}\to *CH_{3}OH/*O\to^{*}OH+CH_{4}$ $\to *+CH_{4}+H_{2}O$. The step determining the rate is $*OH+CH_{4}+H_{2}O+H^{+}+e^{-}\to *+CH_{4}+2H_{2}O$, representing a 1.212 V limiting potential.

The Gibbs free-energy changes for each intermediate involved in Nb-THQ electrocatalytic CO_2_ reduction are depicted in Figure 9b, while the corresponding electrochemical equations and Gibbs free-energy values can be found in Table S8. Similar to Zr-THQ, the adsorption of Nb-THQ on all C_1_ products, except CH_4_, exhibits strong binding that prevents their desorption and subsequent product formation. Therefore, in the analysis of the Nb-THQ electrocatalytic process, we solely focus on the pathway leading to CH_4_ generation. Upon CO_2_ adsorption on the surface of the catalyst, both *COOH and *OCHO formation are exothermic with reduced free energy. In the 2e process, *$COOH\to^{*}CO$, an exothermic free energy-reduced reaction is observed, whereas *$OCHO\to^{*}OCHOH$ requires the overcoming of a 0.196 eV energy barrier (Table S8). Moving on to the 3e process, all possibilities involve heat-absorbing reactions with increasing free energies. The conversion of *$CO\to^{*}COH$ necessitates a 1.265 eV energy barrier (Table S8), while *$OCHOH\to^{*}OCH$ requires a 1.572 eV energy barrier (Table S8). The barrier for *$CO{/}^{*}OCHOH\to^{*}CHO$ is relatively low, at 0.272 eV (Table S8)/0.195 eV (Table S8), resulting in the formation of a *CHO intermediate. Subsequently, protonation steps mostly involve reduced free-energy exothermic reactions, which facilitates their occurrence. In 8e, the final process, a 0.833 eV energy barrier must be overcome. In summary, the primary product of Nb-THQ electrocatalytic CO_2_ reduction is CH_4_. The pathway can be written as follows: $*+CO_{2}\to^{*}COOH{/}^{*}OCHO\to^{*}OCHOH{/}^{*}CO\to^{*}CHO\to^{*}OCH_{2}\to^{*}OCH_{3}\to^{*}O$ $\to^{*}OH+CH_{4}\to *+CH_{4}+H_{2}O$. The step determining the rate is $*OH+CH_{4}+H_{2}O+H^{+}+e^{-}\to *+CH_{4}+2H_{2}O$, representing a 0.883 V limiting potential.

In terms of adsorption energy, Tc-THQ exhibits strong CO adsorption, making it unnecessary to consider CO products in the prediction of its catalytic activity. Figure 9c presents the change in Gibbs free energy of protonation for each intermediate involved in Tc-THQ electrocatalytic CO_2_ reduction, while the corresponding electrochemical reaction equations and changes in Gibbs free energy are provided in Table S9. Upon CO_2_ adsorption on this surface, the 1e step of the protonation process, forming *OCHO/*COOH, is a reduced free-energy exothermic reaction, which facilitates its occurrence. Similarly, the 2e process, which generates *CO/*OCHOH intermediates, also is an exothermic process with reduced energy. Moving to the 3e process, *CO → *CHO/*COH requires the overcoming of 0.422 eV/0.880 eV energy barriers (Table S9), both of which are relatively high. However, *OCHOH → *CHO is a reduced free-energy exothermic reaction, leading to 3e intermediate *CHO. In the 4e process that follows, the conversion of *$CHO\to *OCH_{2}$ requires the crossing of a 0.174 eV energy barrier. In subsequent protonation, the product is obtained by considering HCHO desorption. However, a relatively high 0.801 eV energy barrier must be overcome for the HCHO desorption step to occur. On the other hand, *$OCH_{2}\to^{*}OCH_{3}$ is exothermic, with decreased free energy, resulting in the generation of an *OCH_3_ intermediate in the 5e step. The subsequent protonation steps mostly involve reduced free-energy exothermic reactions until reaching the 8e process. However, the 8e process has to overcome an energy barrier of 0.373 eV. In conclusion, in the CO_2_ electrocatalytic reduction of Tc-THQ, the primary product is CH_4_, and the reaction pathway can be described as follows: $*+CO_{2}\to^{*}OCHO\to^{*}OCHOH\to^{*}CHO\to^{*}OCH_{2}\to^{*}OCH_{3}\to^{*}CH_{3}OH{/}^{*}O+CH_{4}\to^{*}OH+$ $CH_{4}\to^{*}+CH_{4}$. The rate-determining step is *$OH+CH_{4}+H_{2}O+H^{+}+e^{-}\to *+CH_{4}+2H_{2}O$, corresponding to a 0.373 V limiting potential.

Figure 9d illustrates the changes in Gibbs free energy of each electron-transfer step involved in Ru-THQ electrocatalytic CO_2_ reduction. The corresponding electrochemical equation and values for the Gibbs free-energy changes are given in Table S10. As observed in Figure 9d, the 1e and 2e steps predominantly exhibit exothermic reactions with reduced free energies, facilitating the generation of *CO/*OCHOH intermediates. However, the subsequent possible reaction paths, namely *$CO\to^{*}COH$ and *$OCHOH\to^{*}OCH$, require the overcoming of relatively high energy barriers (1.136 eV (Table S10) and 0.992 eV (Table S10), respectively) and are, thus, not considered. With the 3e process, *$OCHOH\to^{*}CHO$ is exothermic, decreases free energy, and occurs readily. Although *$CO\to^{*}CHO$ is an energy-increasing process, the energy barrier of 0.190 eV (Table S10) is a relatively low value to be crossed, allowing for protonation and the formation of the *CHO intermediate. Subsequently, the 4e process from *$CHO\to^{*}OCH_{2}$ involves an energy-enhancing adsorption reaction that requires the overcoming of a 0.358 eV(Table S10) energy barrier. From this point onward, until the completion of the 8e process leading to CH_4_ production, the reactions are predominantly exothermic with decreasing free energy. While the pathway considers HCOOH, HCHO, and CH_3_OH desorption steps requiring the overcoming of 0.540 eV (Table S10), 0.520 eV (Table S10), and 0.527 eV (Table S10) energy barriers, respectively, all these values are higher than the 0.358 eV energy barrier for CH_4_ generation. Therefore, the main product of Ru-THQ electrocatalytic CO_2_ reduction is CH_4_, and reaction pathway can be described as follows: $*+CO_{2}\to^{*}OCHO{/}^{*}COOH\to^{*}OCHOH{/}^{*}CO\to^{*}CHO\to^{*}OCH_{2}\to^{*}OCH_{3}\to^{*}CH_{3}OH{/}^{*}O+CH_{4}$ $\to^{*}OH+CH_{4}\to *+CH_{4}\to *+CH_{4}$. The step determining the rate is *$CHO+H_{2}O+H^{+}+e^{-}\to^{*}OCH_{2}+H_{2}O$, corresponding to a 0.358 V limiting potential.

Figure 9e presents a step diagram depicting changes in Gibbs free energy of each possible intermediate involved in Rh-THQ electrocatalytic reduction of CO_2_. The corresponding electrochemical equations and values for Gibbs free-energy changes are given in Table S11. Upon CO_2_ adsorption on the surface of the catalyst, protonation takes place under external voltage. The production of *OCHO is an adsorptive process with an energy increase, whereas the production of *COOH is exothermic with an energy decrease. Therefore, in the 1e process, *COOH is predominantly produced. In the subsequent 2e process, *COOH → *CO, an energy barrier of 0.208 eV (Table S11) must be overcome. The 4e process of *CHO → *OCH_2_ requires the overcoming of a 0.349 eV (Table S11) energy barrier. All other steps involve reduced free-energy exothermic reactions, facilitating their occurrence until the 8e process concludes with the formation of CH_4_. Although the generation of HCHO and CH_3_OH was considered, the desorption steps for these two products require the overcoming of 0.501 eV (Table S11) and 0.405 eV (Table S11) energy barriers, respectively, both of which are higher than the 0.349 eV energy barrier for CH_4_ generation. Hence, CH_4_ predominates as the main product. In conclusion, Rh-THQ electrocatalytic CO_2_ reduction yields CH_4_ as the primary product, and the pathway can be described as follows: $*+CO_{2}\to^{*}COOH\to^{*}CO\to^{*}CHO\to^{*}OCH_{2}\to^{*}OCH_{3}\to^{*}CH_{3}OH\to^{*}OH+CH_{4}\to *+CH_{4}$. The rate-determining step corresponds to a 0.349 V limiting potential.

### 3.6. Analysis of Electronic Structures

Section 3.5 Gibbs free-energy changes in each step in detail, followed by a discussion of the steps that determine the rate, the limiting potential, and the main products associated with each catalyst. Among the ten studied fourth-period transition metal elements, the predominant reduction product observed for Y-THQ, Ag-THQ, and Cd-THQ is HCOOH. For Mo-THQ, the major product is HCHO. CO is identified as the primary product for Pd-THQ. Lastly, CH_4_ emerges as the main product for Zr, Nb, Tc, Ru, and Rh.

The rate-determining steps, limiting potentials, and overpotentials of the catalytic processes for the ten metals are presented in Table 2. Among these catalysts, Zr-THQ exhibited the highest limiting potential at 1.212 V and the highest overpotential at 1.043 V. Overpotentials for the remaining catalysts ranged from 0.189 V to 0.964 V, which compares favourably with the most active step surfaces, namely Cu(211) (η=0.77 V) and Pt(111) (η=0.46 V) [46]. Based on our theoretical findings, Zr-THQ shows great promise as an electrocatalyst for CO_2_ reduction.

**Figure 10:**
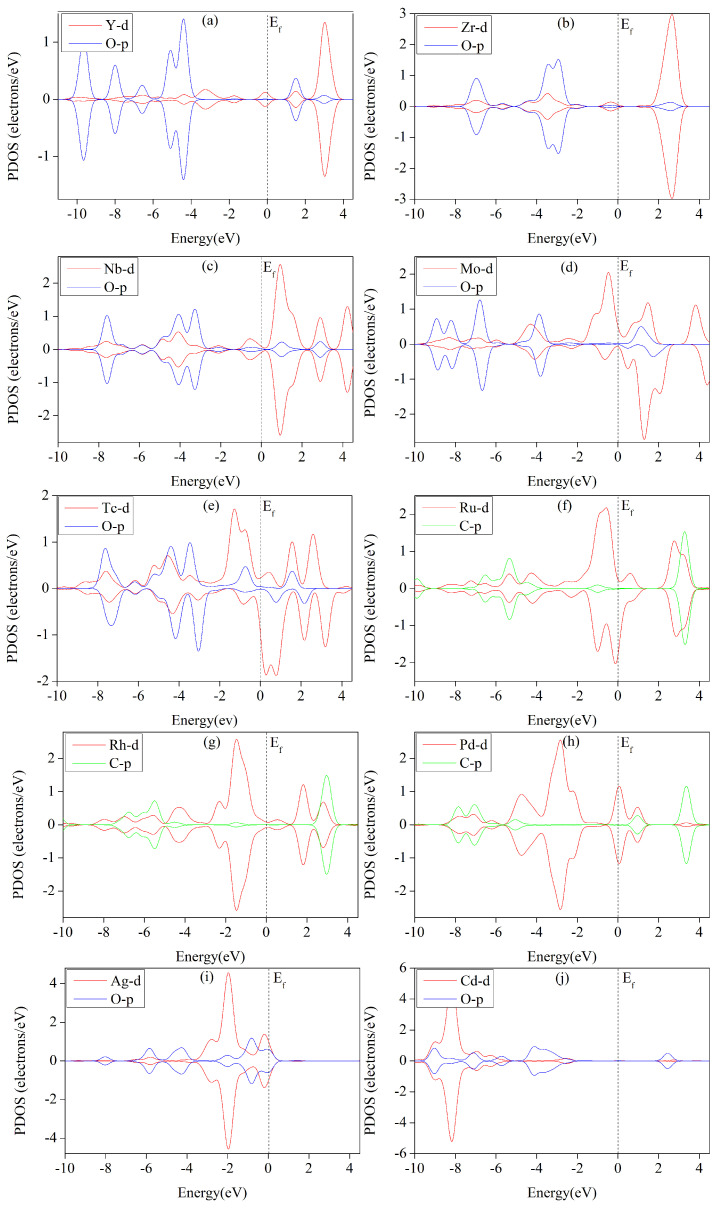
Partition density plots illustrating the predicted adsorption of *OCHOH on Y and Cd; *OH on Zr, Nb, and Tc; *OCH_2_ on Mo; *CHO on Ru and Rh; *COOH on Pd; and *OCHO on Ag. The dashed line indicates the Fermi level. The red, blue, and green lines correspond to metal 4d orbitals, oxygen 2p orbitals, and carbon 2p orbitals, respectively. (**a**) Y-THQ; (**b**) Zr-THQ; (**c**) Nb-THQ; (**d**) Mo-THQ; (**e**) Tc-THQ; (**f**) Ru-THQ; (**g**) Rh-THQ; (**h**) Pd-THQ; (**i**) Ag-THQ; (**j**) Cd-THQ.

The theory of metal–ligand bonding in metalo-organic catalysts shows that interactions between catalyst and intermediates primarily involve σ and π bonds. Figure 10 reveals a distinct overlapping of 4d orbitals in the metal and 2p orbitals in the oxygen (O) or carbon (C) atoms in the critical intermediates (*OCHOH, *OH, *OCH_2_, *CHO, *COOH, or *OCHO), whether they spin up or down. This observation suggests strong binding between TM-THQ (transition metal thioether quinone) and the intermediates. Notably, the d and p orbital overlap effect in Figure 10b–d is more pronounced compared to the others, indicating the interactions of Zr, Nb, and Mo with their respective intermediates are stronger than those of the other catalysts. A stronger interaction leads to greater stability of adsorption intermediates and a higher energy barrier that must be crossed for the catalysis reactions to occur. This results in a greater free-energy increase in the critical step of CO_2_ reduction catalysed by TM-HAB (transition metal heteroatom-bonded). Consequently, the reducing reaction-limiting potential becomes more negative. As shown in Table 2 the limiting potentials ($U_{L}$) for electrocatalytic carbon dioxide reduction using Zr-THQ, Nb-THQ, and Mo-THQ are 1.212 eV, 0.883 eV, and 0.718 eV, respectively, which are higher than for other catalysts. These findings align well with the results obtained from the Partial Density of States (PDOS) analysis.

## 4. Conclusions

This study investigates the electrocatalytic carbon dioxide reduction reaction through the construction of an organometallic network framework using fourth-period transition metal–tetrahydroxybenzoquinone (TM-THQ). Density functional theory simulations show that in a TM-THQ system consisting of 10 monolayers of fourth-period transition metals from Y to Cd, binding energies between metal atoms and THQ are sufficiently strong to stabilise the metal atom dispersion within the THQ framework. Most TM-THQ systems exhibit favourable selectivity towards CO_2_ reduction, with the exception of Tc and Ru, which cannot compete with the hydrogen evolution reaction (HER) in the electrocatalytic CO_2_ reduction process. Tc and Ru require a solution environment with pH > 5.716 and 8.819, respectively, to exhibit CO_2_ reduction selectivity. The main product of Y, Ag, and Cd is HCOOH, while Mo predominantly produces HCHO, Pd generates CO, and Zd yields HCOOH. In contrast, Zr, Nb, Tc, Ru, and Rh primarily produce CH_4_ as the main product. Among these TM-THQ systems, Zr-THQ exhibits the highest limiting potential, at 1.212 V, and the highest overpotential, at 1.043 V. The overpotentials of the other metal–organic frameworks are between 0.179 V and 0.949 V, showing relatively low values. Based on these findings, we predict that TM-THQ frameworks constructed using fourth-period transition metals and tetrahydroxyquinone will exhibit robust activity in the electrocatalytic reduction of carbon dioxide, which makes them promising carbon dioxide reduction electrocatalysts.

## Figures and Tables

**Table 1:** Different structural characterisations of TM-THQ. Analysis of the Hirshfeld charge of a metal atom ($Q_{TM}$) and the nearest O atom ($Q_{O}$), metal atom spin, and the bond distance to the nearest O atom ($R_{TM-O}$).

TM-THQ	$Q_{\mathrm{TM}}$/e	Spin-TM	$Q_{O}$/e	$R_{\mathrm{TM}-O}$/Å
Y	1.0963	0.000	−0.2936	2.235
Zr	1.0686	0.000	−0.2719	2.060
Nb	0.8625	0.000	−0.2487	2.022
Mo	0.7139	2.459	−0.2192	2.034
Tc	0.4140	0.000	−0.1657	2.017
Ru	0.4486	0.000	−0.1808	2.023
Rh	0.3776	0.000	−0.1726	2.028
Pd	0.5272	0.000	−0.2074	2.076
Ag	0.4393	0.000	−0.2046	2.345
Cd	0.6934	0.000	−0.2380	2.254

**Table 2:** Major products, rate-determining steps, limiting potentials ($U_{L}$/V), and overpotentials (η/V) of TM-THQ electrocatalysts for carbon dioxide reduction.

TM-HAB	Major Product	Rate-Determining Step	$U_{L}$	η
Y	HCOOH	*OCHOH→∗+HCOOH	0.699	0.949
Zr	CH_4_	*$OH+CH_{4}+H_{2}O+H^{+}+e^{-}\to *+CH_{4}+2H_{2}O$	1.212	1.043
Nb	CH_4_	*$OH+CH_{4}+H_{2}O+H^{+}+e^{-}\to *+CH_{4}+2H_{2}O$	0.883	0.714
Mo	HCHO	$*OCH_{2}\to *+HCHO$	0.718	0.788
Tc	CH_4_	$*OH+CH_{4}+H_{2}O+H^{+}+e^{-}\to *+CH_{4}+2H_{2}O$	0.373	0.204
Ru	CH_4_	$*CHO+H_{2}O+H^{+}+e^{-}\to *OCH_{2}+H_{2}O$	0.358	0.189
Rh	CH_4_	$*CHO+H_{2}O+H^{+}+e^{-}\to *OCH_{2}+H_{2}O$	0.348	0.179
Pd	CO	$*+CO_{2}+H^{+}+e^{-}\to *COOH$	0.564	0.67
Ag	HCOOH	$*+CO_{2}+H^{+}+e^{-}\to *OCHO$	0.714	0.964
Cd	HCOOH	*OCHOH→∗+HCOOH	0.587	0.837

## Data Availability

No new data were created or analyzed in this study. Data sharing is not applicable to this article.

## Supplementary Materials

The following supporting information can be downloaded at: https://www.mdpi.com/article/10.3390/molecules29102320/s1, Table S1: E_b_ is the binding energy betweenthe TM and the TM–THQ, E_c_ is the cohesive energy of the bulk TM, E_f_ denotes the formation energy of TM-THQ catalysts; Table S2: Gibbs free energy change (ΔG/eV) of the first protonation step in the CO_2_ reduction reaction (CO_2_RR) and H_2_ evolution reaction (HER) on the TM–THQ; Table S3: Adsorption energy of TM-THQ on C_1_ product; Table S4: Pd-THQ electrocatalytic CO_2_ reduction steps for each intermediate and Gibbs free energy variation; Table S5: Mo-THQ electrocatalytic CO_2_ reduction steps for each intermediate and Gibbs free energy variation; Table S6: Y-THQ, Ag-THQ, Cd-T HQ electrocatalytic CO_2_ reduction steps for each intermediate and Gibbs free energy variation; Table S7: Zr-THQ electrocatalytic CO_2_ reduction steps for each intermediate and Gibbs free energy variation; Table S8: Nb-THQ electrocatalytic CO_2_ reduction steps for each intermediate and Gibbs free energy variation; Table S9: Tc-THQ electrocatalytic CO_2_ reduction steps for each intermediate and Gibbs free energy variation; Table S10: Ru-THQ electrocatalytic CO_2_ reduction steps for each intermediate and Gibbs free energy variation. Table S11: Rh-THQ electrocatalytic CO_2_ reduction steps for each intermediate and Gibbs free energy variation; Table S12: Structural modelling of each intermediate in TM-THQ electrocatalytic carbon dioxide reduction; Figure S1: TM-THQ electrocatalytic CO_2_ reduction for each electronic step and reaction pathway. The reaction starts with *+CO_2_, where * denotes the catalyst and the green box is the products [41,42,43,44,45].
