# Supplementary material for: Density Functional Study of Electrocatalytic Carbon Dioxide Reduction in Fourth-Period Transition Metal–Tetrahydroxyquinone Organic Framework
Source: Molecules. 2024 May 15;29(10):2320. doi: 10.3390/molecules29102320 (PMC11123802; doi:10.3390/molecules29102320)
Supplement: Supplementary file 1 [file molecules-29-02320-s001.zip › molecules-2978292-supplementary.pdf]

Density Functional Study of Electrocatalytic Carbon Dioxide Reduction in Fourth-period Transition Metal-Tetrahydroxyquinone Organic Framework

Yufeng Wen[1], Xianshi Zeng[1], Yanan Xiao[1], Wen Ruan[1], Kai Xiong[2,3] , Zhangli Lai[1*]

1 School of Mathematical Sciences and Physics, Jinggangshan University, Ji'an 343009, China; jgsuwyf@sina.com(Y,W); zengxueliang@163.com(X,Z); yananxiao06@163.com(Y,X) ; ruanwen@jgsu.edu.cn(W,R)

2 Materials Genome Institute, National Center for International Research on Photoelectric and Energy Materials, School of Materials and Energy, Yunnan University, Kunming 650091, China; xiongkai@ynu.edu.cn(K,X)

3 Advanced Computing Center, Information Technology Center, Yunnan University, Kunming 650091, China.

* Correspondence: laizhangli@jgsu.edu.cn

Table S. 1 E_b is the binding energy betweenthe TM and the TM–THQ, E_c is the cohesive energy of the bulk TM, E_f denotes the formation energy of TM-THQ catalysts.

TM	E_b(eV)	E_c(eV)	E_f(eV)
Y	-11.100	-5.823	-25.382
Zr	-13.295	-7.918	-20.721
Nb	-10.145	-8.576	-14.119
Mo	-8.821	-7.633	-7.894
Tc	-7.820	-8.222	-8.876
Ru	-7.113	-7.968	-9.530
Rh	-6.047	-7.435	-9.366
Pd	-4.354	-4.592	-9.784
Ag	-3.130	-4.192	-7.835
Cd	-3.163	-1.118	-15.976

Table S. 2 Gibbs free energy change (ΔG/eV) of the first protonation step in the CO_2 reduction reaction (CO_2RR) and H_2 evolution reaction (HER) on the TM–THQ

TM	**ΔG[*H]**	**ΔG*COOH**	**ΔG*OCHO**
Y	0.777	0.981	-0.471
Zr	-0.151	2.168	-1.087
Nb	-0.436	-0.448	-1.067
Mo	-0.347	-0.130	-0.409
Tc	-0.513	-0.476	-0.174
Ru	-0.516	-0.658	0.008
Rh	-0.323	-0.233	0.686
Pd	0.669	0.564	1.120
Ag	1.054	0.731	0.715
Cd	0.766	0.882	0.384

Table S. 3 Adsorption energy of TM-THQ on C_1 product

**TM**	CO	HCOOH	HCHO	CH_3OH	CH_4
Y	-0.503	-0.807	-0.654	-0.916	-0.107
Zr	-1.019	-1.213	-1.186	-1.352	-0.301
Nb	-1.559	-1.537	-1.219	-1.085	-0.093
Mo	-1.597	-0.926	-0.808	-1.013	-0.466
Tc	-1.109	-0.811	-0.999	-0.973	-0.156
Ru	-1.987	-0.766	-0.821	-0.856	-0.287
Rh	-1.031	-0.456	-0.606	-0.714	-0.358
Pd	-0.247	-0.213	-0.269	-0.357	-0.164
Ag	-0.671	-0.596	-0.659	-0.655	-0.542
Cd	-0.537	-0.702	-0.663	-0.815	-0.364

Table S. 4 Pd-THQ electrocatalytic CO_2 reduction steps for each intermediate and Gibbs free energy variation.

n(H^++e^-)transferred	Chemical reaction equation	ΔG
1	$* + CO_2 + H^+ + e^- \rightarrow *COOH$	0.564
	$* + CO_2 + H^+ + e^- \rightarrow *OCHO$	1.120
2	$*COOH + H^+ + e^- \rightarrow *CO + H_2O$	-0.300
3	$*CO + H_2O + H^+ + e^- \rightarrow *CHO + H_2O$	0.734
	$*CO + H_2O + H^+ + e^- \rightarrow *COH + H_2O$	1.394
	$*CO + H_2O \rightarrow * + CO + H_2O$	0.387

Table S. 5 Mo-THQ electrocatalytic CO_2 reduction steps for each intermediate and Gibbs free energy variation。

$n(H^+ + e^-)$transferred	Chemical reaction equation	ΔG
1	$* + CO_2 + H^+ + e^- \rightarrow *COOH$	-0.130
	$* + CO_2 + H^+ + e^- \rightarrow *OCHO$	-0.409
2	$*COOH + H^+ + e^- \rightarrow *CO + H_2O$	-0.291
	$*OCHO + H^+ + e^- \rightarrow *OCHOH$	-0.251
3	$*CO + H_2O + H^+ + e^- \rightarrow *CHO + H_2O$	0.475
	$*CO + H_2O + H^+ + e^- \rightarrow *COH + H_2O$	0.805
	$*OCHOH + H^+ + e^- \rightarrow *CHO + H_2O$	0.713
	$*OCHOH + H^+ + e^- \rightarrow *OCH + H_2O$	2.323
	$*OCHOH \rightarrow * + HCOOH$	1.005
4	$*CHO + H_2O + H^+ + e^- \rightarrow *OCH_2 + H_2O$	-0.404
	$*COH + H_2O + H^+ + e^- \rightarrow *C + 2H_2O$	1.595
5	$*OCH_2 + H_2O + H^+ + e^- \rightarrow *OCH_3 + H_2O$	-1.038
	$*OCH_2 + H_2O + H^+ + e^- \rightarrow * + HCHO + H_2O + H^+ + e^-$	0.718
6	$*OCH_3 + H_2O + H^+ + e^- \rightarrow *CH_3OH + H_2O$	0.139
	$*OCH_3 + H_2O + H^+ + e^- \rightarrow *O + CH_4 + H_2O$	-1.229
7	$*O + CH_4 + H_2O + H^+ + e^- \rightarrow *OH + CH_4 + H_2O$	0.043
	$*CH_3OH + H_2O + H^+ + e^- \rightarrow *OH + CH_4 + H_2O$	-1.326
8	$*OH + CH_4 + H_2O + H^+ + e^- \rightarrow * + CH_4 + 2H_2O$	0.853

Table S. 6 Y-THQ、Ag-THQ、Cd-THQ electrocatalytic CO_2 reduction steps for each intermediate and Gibbs free energy variation。

catalysts	$n(H^+ + e^-)$transferred	Chemical reaction equation	ΔG
Y-THQ	1	$* + CO_2 + H^+ + e^- \rightarrow *COOH$	0.981
		$* + CO_2 + H^+ + e^- \rightarrow *OCHO$	-0.471
	2	$*OCHO + H^+ + e^- \rightarrow *OCHOH$	0.138
	3	$*OCHOH + H^+ + e^- \rightarrow *CHO + H_2O$	1.471
		$*OCHOH + H^+ + e^- \rightarrow *OCH + H_2O$	1.576
		$*OCHOH \rightarrow * + HCOOH$	0.699
Ag-THQ	1	$* + CO_2 + H^+ + e^- \rightarrow *COOH$	0.731
		$* + CO_2 + H^+ + e^- \rightarrow *OCHO$	0.714
	2	$*OCHO + H^+ + e^- \rightarrow *OCHOH$	-1.337
	3	$*OCHOH + H^+ + e^- \rightarrow *CHO + H_2O$	1.212
		$*OCHOH + H^+ + e^- \rightarrow *OCH + H_2O$	1.349
		$*OCHOH \rightarrow * + HCOOH$	0.546
Cd-THQ	1	$* + CO_2 + H^+ + e^- \rightarrow *COOH$	0.882
		$* + CO_2 + H^+ + e^- \rightarrow *OCHO$	0.384
	2	$*OCHO + H^+ + e^- \rightarrow *OCHOH$	-0.771
	3	$*OCHOH + H^+ + e^- \rightarrow *CHO + H_2O$	1.077
		$*OCHOH + H+ + e- \rightarrow *OCH + H_2O$	0.968
		$*OCHOH \rightarrow * + HCOOH$	0.587

Table S. 7 Zr-THQ electrocatalytic CO_2 reduction steps for each intermediate and Gibbs free energy variation。

n(H^++e^-)transferred	Chemical reaction equation	ΔG
1	* + CO_2 +H^+ + e- →*COOH	2.167
	* + CO_2+ H^+ + e^- → *OCHO	-1.086
2	*OCHO + H^+ + e^- → *OCHOH	0.269
3	*OCHOH + H^+ + e^- → *CHO + H_2O	1.083
	*OCHOH + H^+ + e^- → *OCH + H_2O	2.054
4	*CHO + H_2O + H^+ + e^- → *OCH_2 + H_2O	-0.988
5	*OCH_2 + H_2O + H^+ + e^- → *OCH_3 + H_2O	-1.211
6	*OCH_3 + H_2O + H^+ + e^- → *CH_3OH + H_2O	0.087
	*OCH_3 + H_2O + H^+ + e^- → *O + CH_4 + H_2O	-0.103
7	*O + CH_4 + H_2O + H^+ + e^- → *OH + CH_4 + H_2O	-1.069
	*CH_3OH + H_2O + H^+ + e^- → *OH + CH_4 + H_2O	-1.261
8	*OH + CH_4 + H_2O + H^+ + e^- → * + CH_4 + 2H_2O	1.212

Table S. 8 Nb-THQ electrocatalytic CO_2 reduction steps for each intermediate and Gibbs free energy variation。

n(H^++e^-)transferred	Chemical reaction equation	ΔG
1	* + CO_2 + H^+ + e^- →*COOH	-0.447
	* + CO_2 + H^+ + e^- → *OCHO	-1.066
2	*COOH + H^+ + e^- → *CO + H_2O	-0.499
	*OCHO + H^+ + e^- → *OCHOH	0.196
3	*CO + H_2O + H^+ + e^- → *CHO + H_2O	0.272
	*CO + H_2O + H^+ + e^- → *COH + H_2O	1.265
	*OCHOH + H^+ + e^- → *CHO + H_2O	0.195
	*OCHOH + H^+ + e^- → *OCH + H_2O	1.572
4	*CHO + H_2O + H^+ + e^- → *OCH_2 + H_2O	-0.341
5	*OCH_2 + H_2O + H^+ + e^- → *OCH_3 + H_2O	-1.244
6	*OCH_3 + H_2O + H^+ + e^- → *CH_3OH + H_2O	0.398
	*OCH_3 + H_2O + H^+ + e^- → *O + CH_4 + H_2O	-0.982
7	*O + CH_4 + H_2O + H^+ + e^- → *OH + CH_4 + H_2O	-0.264
8	*OH + CH_4 + H_2O + H^+ + e^- → * + CH_4 + 2H_2O	0.883

Table S. 9 Tc-THQ electrocatalytic CO_2 reduction steps for each intermediate and Gibbs free energy variation。

n(H^++e^-)transferred	Chemical reaction equation	ΔG
1	$* + CO_2 + H^+ + e^- \rightarrow *COOH$	-0.475
	$* + CO_2 + H^+ + e^- \rightarrow *OCHO$	-0.174
2	$*COOH + H^+ + e^- \rightarrow *CO + H_2O$	-0.613
	$*OCHO + H^+ + e^- \rightarrow *OCHOH$	-0.357
3	$*CO + H_2O + H^+ + e^- \rightarrow *CHO + H_2O$	0.422
	$*CO + H_2O + H^+ + e^- \rightarrow *COH + H_2O$	0.880
	$*OCHOH + H^+ + e^- \rightarrow *CHO + H_2O$	-0.135
	$*OCHOH + H^+ + e^- \rightarrow *OCH + H_2O$	1.822
	$*OCHOH \rightarrow * + HCOOH$	0.664
4	$*CHO + H_2O + H^+ + e^- \rightarrow *OCH_2 + H_2O$	0.174
5	$*OCH_2 + H_2O + H^+ + e^- \rightarrow *OCH_3 + H_2O$	-0.761
	$*OCH_2 + H_2O + H^+ + e^- \rightarrow * + HCHO + H_2O + H^+ + e^-$	0.801
6	$*OCH_3 + H_2O + H^+ + e^- \rightarrow *CH_3OH + H_2O$	-0.168
	$*OCH_3 + H_2O + H^+ + e^- \rightarrow *O + CH_4 + H_2O$	-0.646
7	$*O + CH_4 + H_2O + H^+ + e^- \rightarrow *OH + CH_4 + H_2O$	-0.376
	$*CH_3OH + H_2O + H^+ + e^- \rightarrow *OH + CH_4 + H_2O$	-0.855
8	$*OH + CH_4 + H_2O + H^+ + e^- \rightarrow * + CH_4 + 2H_2O$	0.373

Table S. 10 Ru-THQ electrocatalytic CO_2 reduction steps for each intermediate and Gibbs free energy variation。

$n(H^{+}+e^{-})$transferred	Chemical reaction equation	ΔG
1	$* + CO_2 + H^+ + e^- \rightarrow *COOH$	-0.658
	$* + CO_2 + H^+ + e^- \rightarrow *OCHO$	0.008
2	$*COOH + H^+ + e^- \rightarrow *CO + H_2O$	-0.589
	$*OCHO + H^+ + e^- \rightarrow *OCHOH$	-0.624
3	$*CO + H_2O + H^+ + e^- \rightarrow *CHO + H_2O$	0.190
	$*CO + H_2O + H^+ + e^- \rightarrow *COH + H_2O$	1.136
	$*OCHOH + H^+ + e^- \rightarrow *CHO + H_2O$	-0.440
	$*OCHOH + H^+ + e^- \rightarrow *OCH + H_2O$	0.992
	$*OCHOH \rightarrow * + HCOOH$	0.540
4	$*CHO + H_2O + H^+ + e^- \rightarrow *OCH_2 + H_2O$	0.358
5	$*OCH_2 + H_2O + H^+ + e^- \rightarrow *OCH_3 + H_2O$	-0.448
	$*OCH_2 + H_2O + H^+ + e^- \rightarrow * + HCHO + H_2O + H^+ + e^-$	0.520
6	$*OCH_3 + H_2O + H^+ + e^- \rightarrow *CH_3OH + H_2O$	-0.514
	$*OCH_3 + H_2O + H^+ + e^- \rightarrow *O + CH_4 + H_2O$	-0.073
7	$*O + CH_4 + H_2O + H^+ + e^- \rightarrow *OH + CH_4 + H_2O$	-1.029
	$*CH_3OH + H_2O + H^+ + e^- \rightarrow *OH + CH_4 + H_2O$	-0.587
	$*CH_3OH + H_2O \rightarrow * + CH_3OH + H_2O$	0.527
8	$*OH + CH_4 + H_2O + H^+ + e^- \rightarrow * + CH_4 + 2H_2O$	-0.333

Table S. 11 Rh-THQ electrocatalytic CO_2 reduction steps for each intermediate and Gibbs free energy variation。

n(H^++e^-)transferred	Chemical reaction equation	ΔG
1	* + CO_2 + H^+ + e^- →*COOH	-0.233
	* + CO_2+ H^+ + e^- → *OCHO	0.686
2	*COOH + H^+ + e^- → *CO + H_2O	0.208
3	*CO + H_2O + H^+ + e^- → *CHO + H_2O	-0.496
	*CO + H_2O + H^+ + e^- → *COH + H_2O	1.169
4	*CHO + H_2O + H^+ + e^- → *OCH_2 + H_2O	0.348
5	*OCH_2 + H_2O + H^++ e^- → *OCH_3+ H_2O	-0.006
	*OCH_2 + H_2O + H^+ + e^- → * + HCHO + H_2O + H^+ + e^-	0.510
6	*OCH_3 + H_2O + H^+ + e^- → *CH_3OH + H_2O	-1.041
	*OCH_3 + H_2O + H^+ + e^- → *O + CH_4 + H_2O	0.589
7	*CH_3OH + H_2O + H^+ + e^- → *OH + CH_4 + H_2O	-0.050
	*CH_3OH + H_2O → * + CH_3OH + H_2O	0.405
8	*OH + CH_4 + H_2O + H^+ + e^- → * + CH_4 + $2H_2O$	-0.824

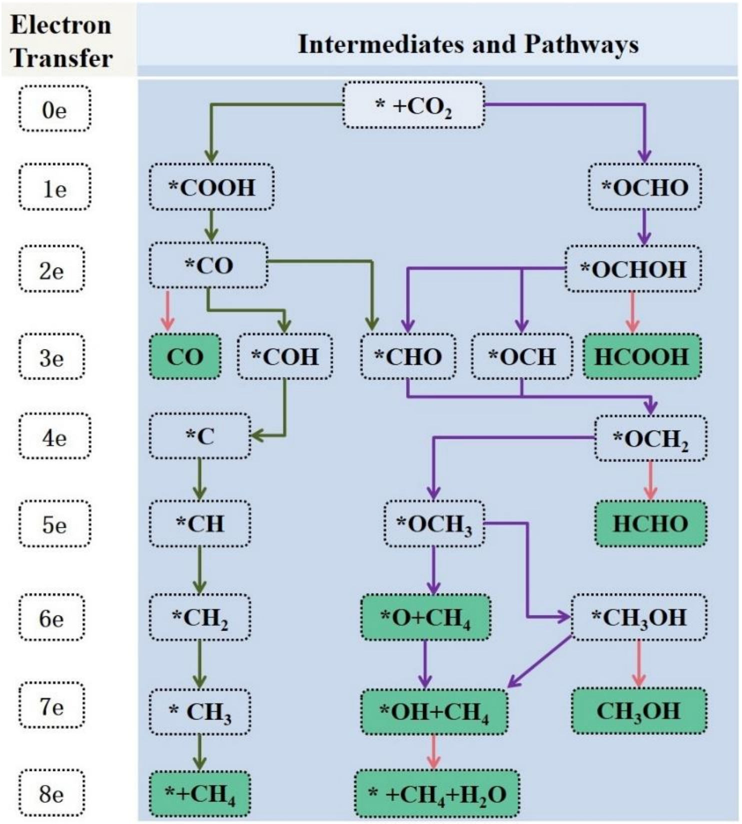

Figure S 1 TM-THQ electrocatalytic CO_2 reduction for each electronic step and reaction pathway. The reaction starts with *+CO_2, where * denotes the catalyst and the green box is the products[1-5].

Table S. 12 Structural modelling of each intermediate in TM-THQ electrocatalytic carbon dioxide reduction.

n(H^++e^-) transferred	Intermediates for each electron transfer step		
1	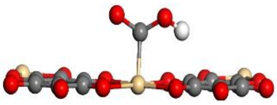	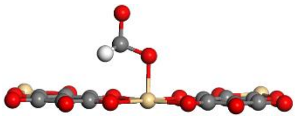	
	*COOH	*OCHO	
2	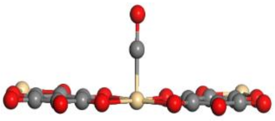	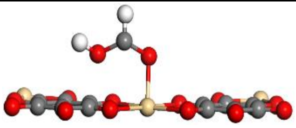	
	*CO	*OCHOH	
3	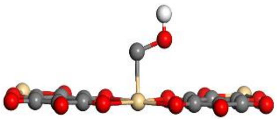	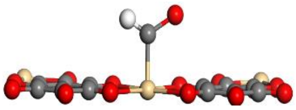	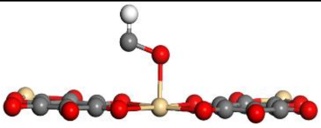
	*COH	*CHO	*OCH
4	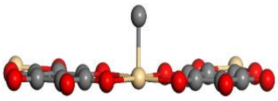	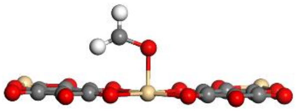	
	*C	*OCH_2	
5	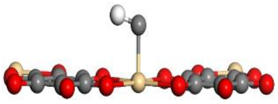	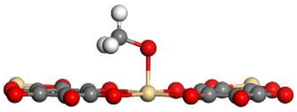	
	*CH	*OCH_3	
6	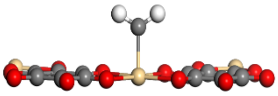	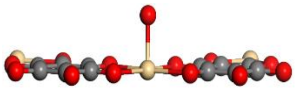	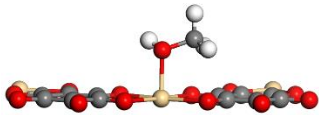
	*CH_2	*O	*CH_3OH
7	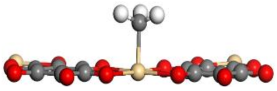	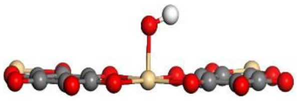	
	*CH_3	*OH	

Reference

[1] Zeng, X.; Liao, L.; Wang, M.; Wang, H. Rare-earth metal-N6 centers in porous carbon for electrocatalytic CO_2 reduction. Physical chemistry chemical physics : PCCP **2023**, 25, 20381–20394

[2] Liu, J.H.; Yang, L.; Ganz, E. Electrocatalytic reduction of CO_2 by two-dimensional transition metal porphyrin sheets. Journal of Materials Chemistry A **2019**, 7, 11944–11952.

[3] Zeng, X.; Liao, L.; Wang, M.; Wang, H. Density functional calculation of two-dimensional transition

metal-hexaiminotriphenylene (TM-HITP) electrocatalytic CO_2 reduction. Catalysis Science Technology **2023**, pp. 5351–5364

[4] Zeng, X.; Liao, L.; Yu, Q.; Wang, M.; Wang, H. Theoretical Prediction of Electrocatalytic Reduction of CO_2 Using a 2D Catalyst Composed of 3d Transition Metal and Hexaamine Dipyrazino Quinoxaline. Chemistry **2023**, p. e202302232

[5] Zeng, X.; Tu, Z.J.; Yuan, Y.; Liao, L.; Xiao, C.; Wen, Y.; Xiong, K. Two-Dimensional Transition Metal-Hexaaminobenzene Monolayer Single-Atom Catalyst for Electrocatalytic Carbon Dioxide Reduction. Nanomaterials **2022**, 12, 4005.
